# Fractal Analysis of Electrodermal Activity for Emotion Recognition: A Novel Approach Using Detrended Fluctuation Analysis and Wavelet Entropy

**DOI:** 10.3390/s24248130

**Published:** 2024-12-19

**Authors:** Luis R. Mercado-Diaz, Yedukondala Rao Veeranki, Edward W. Large, Hugo F. Posada-Quintero

**Affiliations:** 1 Department of Biomedical Engineering, University of Connecticut, Storrs, CT 06269, USA; luis.mercado_diaz@uconn.edu (L.R.M.-D.); yedukondala_rao.veeranki@uconn.edu (Y.R.V.); 2 Department of Electronics and Communication Engineering, Indian Institute of Information Technology Dharwad, Dharwad 580009, India; 3 Department of Psychological Sciences, University of Connecticut, Mansfield, CT 06269, USA; edward.large@uconn.edu

**Keywords:** electrodermal activity, emotional states, fractal analysis, machine learning, detrended fluctuation analysis

## Abstract

The field of emotion recognition from physiological signals is a growing area of research with significant implications for both mental health monitoring and human–computer interaction. This study introduces a novel approach to detecting emotional states based on fractal analysis of electrodermal activity (EDA) signals. We employed detrended fluctuation analysis (DFA), Hurst exponent estimation, and wavelet entropy calculation to extract fractal features from EDA signals obtained from the CASE dataset, which contains physiological recordings and continuous emotion annotations from 30 participants. The analysis revealed significant differences in fractal features across five emotional states (neutral, amused, bored, relaxed, and scared), particularly those derived from wavelet entropy. A cross-correlation analysis showed robust correlations between fractal features and both the arousal and valence dimensions of emotion, challenging the conventional view of EDA as a predominantly arousal-indicating measure. The application of machine learning for emotion classification using fractal features achieved a leave-one-subject-out accuracy of 84.3% and an F1 score of 0.802, surpassing the performance of previous methods on the same dataset. This study demonstrates the potential of fractal analysis in capturing the intricate, multi-scale dynamics of EDA signals for emotion recognition, opening new avenues for advancing emotion-aware systems and affective computing applications.

## 1. Introduction

The recognition of emotional states has emerged as a crucial area of research due to its significant implications in various domains, including mental health, human–computer interaction, and assistive technologies [1,2,3]. Emotions are a fundamental aspect of human experience, influencing communication, perception, and the ability to perform essential adaptive tasks [2,3]. The prevalence of anxiety and depressive disorders, which affected 970 million people worldwide in 2019, highlights the necessity for the development of efficacious methods for the early intervention and prevention of mental health problems [4,5]. Wearable devices have gained considerable attention as a promising platform for the recognition of emotional states. They have the potential to facilitate continuous, non-invasive monitoring of the physiological signals that reflect underlying emotional processes. Among these physiological signals, electrodermal activity (EDA), derived from skin conductance, has emerged as a particularly relevant and informative measure, as evidenced by the literature [6,7]. The analysis of EDA signals has traditionally relied on time-domain, frequency-based, and time-frequency techniques [8]. These approaches have yielded encouraging outcomes; however, they are constrained in their ability to fully capture the intricate nuances of the emotional states–signal relationship [9]. Recently, nonlinear signal processing of EDA signals have been used for emotion recognition [10]. Although these nonlinear methods have demonstrated potential in enhancing the comprehension of emotional states–signal relationships and improving the precision of emotion classification models, the selection of suitable parameters, such as the embedding dimension, delay time, or threshold values, is often necessary to ensure optimal results. Fractal analysis has emerged as a valuable tool for examining intricate, self-similar patterns in time series data [11]. Although traditional methods for analyzing EDA signals have shown promise, they face several key challenges: the complex non-linear nature of physiological responses to emotions, the need for interpretable features that can be linked to underlying physiological processes, and the computational efficiency required for real-world applications. These challenges motivate our exploration of fractal analysis as a novel approach to emotion recognition. In this study, we put forth a novel approach based on fractal analysis for the detection of emotional states using EDA signals.

EDA refers to the changes in the electrical conductivity of the skin, which are modulated by the activity of the sympathetic nervous system [12]. The sympathetic nervous system plays a pivotal role in regulating physiological arousal and emotional responses, rendering EDA a valuable indicator of emotional states [13,14]. The EDA signal is composed of two principal components: the tonic component, which reflects the slow-varying baseline conductivity, and the phasic component, which represents rapid bursts of activity linked to stimulus processing and cognitive load [15,16]. The morphology, amplitude, and timing of the phasic component of the skin conductance response (SCR) provide valuable insights into emotional and cognitive states [12]. However, the complex nonstationary and nonlinear nature of EDA signals poses challenges in extracting reliable information and fully unraveling the intricate relationships between emotional states and the signal characteristics [9].

The time-domain analysis, which extracts features such as mean, variance, skewness, and kurtosis, is capable of quantifying overall trends and variations. However, it may be deficient in its ability to detect abrupt or nonstationary changes and lacks the frequency information that could provide insight into sympathetic dynamics [17,18]. Techniques based on frequency, such as the Fourier transform and the wavelet transform, permit the characterization of frequency bands; however, they may not fully capture the time-varying nature of EDA signals [19]. Time-frequency methods, such as the short-time Fourier transform, provide a combined representation; however, this is accompanied by a reduction in resolution due to the inherent time-frequency trade-off [20].

To address these limitations, advanced nonlinear signal processing methods have been proposed for the automatic recognition of emotions using EDA signals. These methods include isaxEDA, a symbolic approximation-based approach that captures the dynamics of EDA signals [10]; comEDA, which measures the degree of irregularity and unpredictability in the signal [21]; and topEDA, which uncovers the structural properties of EDA using phase space representation of the signal [22]. Network theory-inspired methods, such as netEDA, have been employed to model the intricate interactions and dependencies within EDA signals [22]. In the case of netEDA, the signal is transformed into a network representation, wherein each data point is regarded as a node, and the connections between nodes are determined based on their similarity or temporal proximity. A recent study utilized graph signal analysis to construct an EDA-Graph for automatic emotion detection [23].

Fractal analysis has emerged as a powerful tool for the study of complex, self-similar patterns in time series data [11]. Fractals are geometric objects that exhibit self-similarity across different scales, meaning that their structure appears similar when viewed at different magnifications [24]. A number of physiological signals, including EDA, have been demonstrated to possess fractal properties, reflecting the intrinsic complexity and multiscale organization of the physiological systems [25]. Detrended fluctuation analysis (DFA) is a widely used method for quantifying the fractal properties of time series data [26]. In DFA, the scaling exponent, obtained from the slope of the log–log plot of the average fluctuation versus the segment size, provides a measure of the fractal properties of the signal [27]. It is hypothesized that the application of DFA to EDA signals offers several advantages. First, DFA is robust to non-stationarities and trends in the data, making it suitable for analyzing complex physiological signals that may exhibit long-range correlations and nonlinear dynamics [28]. Secondly, DFA is capable of capturing the multiscale properties of EDA signals, thereby revealing the presence of fractal structures that may be associated with different emotional states [29]. Thirdly, the scaling exponent obtained from DFA provides a quantitative measure of the fractal properties, thus enabling the development of automated emotion classification algorithms based on these features [30].

Our study introduces a novel approach based on fractal analysis, specifically using DFA, for detecting emotional states from EDA signals. By elucidating the fractal properties of EDA signals and identifying discrete fractal signatures correlated with distinct emotional states, our objective is to enhance the accuracy and robustness of emotion recognition algorithms. The proposed approach offers a novel dimension for characterizing emotional responses and has the potential to advance the field of affective computing and the development of emotionally intelligent wearable systems.

## 2. Materials and Methods

### 2.1. Dataset and Emotional States Labeling

For this study, EDA signals were obtained from the “Continuously Annotated Signals of Emotion (CASE)” database, a publicly accessible multimodal dataset specifically designed for emotion recognition research [31,32,33]. The CASE database comprises physiological signals (electrocardiogram, blood volume pressure, electromyography, and EDA) recorded from 30 participants (15 males and 15 females, ages 18–35 years) while they viewed a series of emotionally evocative video stimuli. The video stimuli were selected with the objective of eliciting distinct emotional states. The duration of each video clip was approximately 2–3 min, and the order of presentation was randomized across participants to mitigate potential order effects and carry-over influences from previous videos. It is noteworthy that a two-minute neutral baseline period (blue screen) was incorporated between each video clip, allowing participants to return to a neutral emotional state before proceeding to the subsequent video.

**Figure 1:**
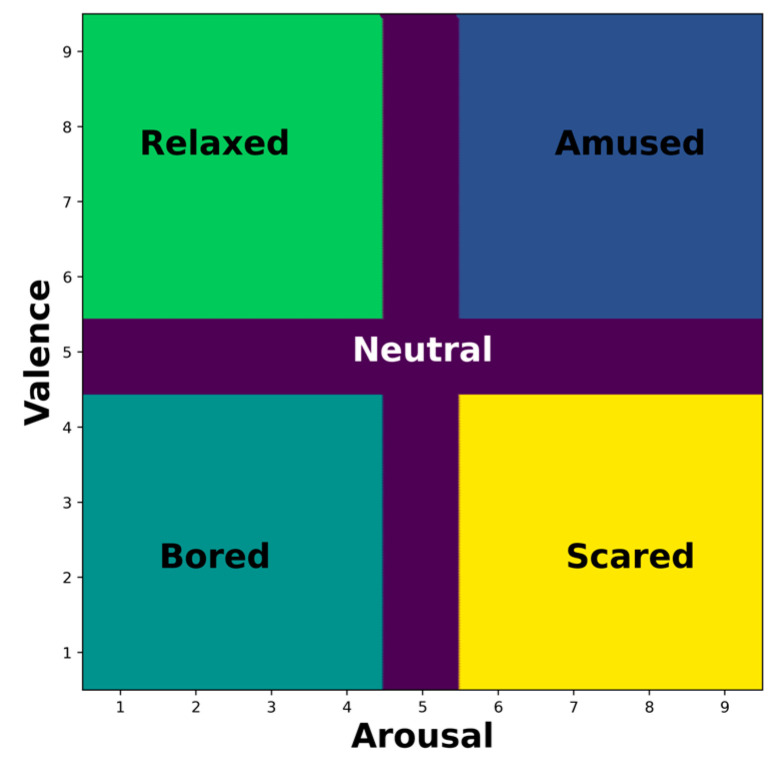
The circumplex model of emotions.

During the experiment, participants were instructed to provide continuous annotations of their experienced emotional states by rating their affective dimensions, valence, and arousal in real time on a scale ranging from 0.5 to 9.5 (see Figure 1). Valence refers to the degree of positive or negative affect, whereas arousal is the degree of calmness or excitement that is measured. The bi-dimensional or circumplex model provides a practical representation of emotional states [34,35]. The continuous annotation of valence and arousal permitted the capture of dynamic emotional responses as they unfolded over time, as opposed to relying on a single retrospective rating for each video clip.

To align the study with a structured emotional framework and facilitate comparative analyses, discrete emotional state labels were derived from specific intervals of the continuously reported arousal and valence scores. This relabeling process yielded five distinct emotional categories, neutral, amused, bored, relaxed, and scared, as shown in Figure 1. Specifically, the ‘neutral’ emotional state was defined as a range of arousal and valence scores from 4.5 to 5.5 on the 9-point scale, creating a clear demarcation for this often-ambiguous central region of the circumplex model. The five discrete emotional categories were derived from specific intervals of continuously reported arousal and valence scores. This approach aligns with the works of [36,37], which emphasized the importance of including the neutral state in emotional process analyses. The ‘neutral’ state was specifically defined within the range of 4.5 to 5.5 on the 9-point scale for both arousal and valence dimensions, providing a clear demarcation of this central region in the circumplex model. This systematic categorization ensures comprehensive coverage of the emotional spectrum while maintaining scientific rigor in our analysis.

The five categories were employed as labels for the machine learning classification analysis. It is important to note that the discrete emotional state labels derived in this study do not necessarily reflect the intended emotions of the video stimuli in the CASE dataset. Rather, they represent the actual emotional experiences reported by the participants, as captured through their continuous arousal and valence ratings. This approach ensures that the analysis accurately represents the emotions experienced by the subjects, rather than relying solely on the emotions intended to be elicited by the video stimuli.

### 2.2. Signal Processing and Fractal Analysis

The raw EDA signals were subjected to preprocessing to eliminate artefacts and high-frequency noise through the utilization of linear filtering techniques [6]. However, EDA signals are characterized by intricate nonlinear and nonstationary attributes, which may not be adequately represented by conventional linear methodologies [13]. To overcome this shortcoming, fractal analysis, a nonlinear signal processing approach, was employed to quantify the intrinsic complexity and multiscale dynamics of EDA signals linked to diverse emotional states.

#### 2.2.1. Linear Preprocessing

A 4th order Butterworth low-pass filter with a cutoff frequency of 60 Hz was applied as shown in Equation (1).

(1) $$y\left[n\right]=\sum_{k=0}^{N}b_{k}x\left[n-k\right]-\sum_{k=1}^{N}a_{k}y\left[n-k\right]$$

where $x\left[n\right]$ is the input EDA signal, $y\left[n\right]$ is the filtered output, and $b_{k}$ and $a_{k}$ are the Butterworth filter coefficients.

#### 2.2.2. Windowing and Detrending

The signals were segmented into overlapping windows of fixed length (e.g., 10 s) with varying overlap percentages (0%, 25%, 50%, and 75%). Within each window, the EDA signal segment was detrended using a Savitzky–Golay filter to remove low-frequency trends and baseline wander. The filter order was set to 3, and the window size was 99 samples, as shown in Equation (2).

(2) $$y_{d}\left[n\right]=\sum_{m=-M}^{M}c_{m}x\left[n+m\right]$$

where $y_{d}\left[n\right]$ is the detrended signal, $x\left[n\right]$ is the input signal segment, $c_{m}$ is the Savitzky–Golay filter coefficient, and 2M+1 is the filter window size.

The 3 s duration window size with 50% overlap was selected after extensive testing of various window configurations (1–10 s, with overlap rates of 0%, 25%, 50%, and 75%). This configuration provided an optimal balance between temporal resolution and feature stability. The 3 s duration aligns with the typical latency of EDA responses (1–4 s) reported in the literature [6,38], while the 50% overlap ensures smooth tracking of temporal changes without losing significant information. We conducted sensitivity analyses that showed our results remained robust within a window size range of 2–4 s, though performance declined significantly outside this range.

#### 2.2.3. Fractal Analysis

Fractal analysis comprises a range of techniques for quantifying the fractal scaling properties and intrinsic complexity of time series data, which can provide valuable insights into the underlying dynamics and irregularities of the signal. In this study, three different fractal analysis techniques were employed: DFA, Hurst exponent estimation, and wavelet entropy calculation.

1. DFA:

DFA [30] is a nonlinear technique for quantifying the fractal scaling properties and intrinsic complexity of time series data. It was employed to analyze the detrended EDA signal segments and extract fractal features, such as scaling exponents and crossover scales.

The DFA method consists of the following steps:

(a) The detrended signal $y_{d}\left[n\right]$ of length N is integrated to obtain the cumulative sum $Y\left(k\right)$ as shown below in Equation (3).
  (3) $$Y\left(k\right)=\sum_{i=1}^{k}\left[signal\left(i\right)-\bar{signal}\right],\;\;k=1,2,\ldots ,L$$
  where $\bar{signal}\;$ is the mean of the signal, and *L* is the signal length.
(b) The integrated series $Y\left(k\right)$ is divided into non-overlapping segments of equal length n, where n is chosen logarithmically spaced scales from 1 to L/4. In each segment, the local trend is estimated by least-squares regression fitting of a linear polynomial of order 1 as $Y_{fit}\left(k\right)$. The fitted polynomial is then subtracted from the segment to obtain the detrended fluctuation $F\left(n,v\right)$ below:
  (4) $$F\left(n,v\right)=\sqrt{\frac{1}{n}\sum_{k=1}^{n}{\left[Y\left(\left(v-1\right)n+k\right)-Y_{fit}\left(k\right)\right]}^{2}}\;$$
  where v represents the segment index, and $Y_{fit}\left(k\right)$ is the m-th order polynomial fit in the v-th segment.
(c) The root mean square of the detrended fluctuations $F\left(n,v\right)$ is calculated over all segments of length n to obtain the average fluctuation function $F\left(n\right)$:
  (5) $$F\left(n\right)=\sqrt{\frac{1}{N_{n}\sum_{v=1}^{N_{n}}F{\left(n,v\right)}^{2}}}$$
  where $N_{n}$ is the number of segments of length n.
(d) This procedure is repeated over a range of segment lengths n to characterize the detrended fluctuations at different time scales.
(e) If the average fluctuation function $F\left(n\right)$ against the segment length n on log–log scales exhibits fractal scaling behavior, the relationship between $F\left(n\right)$ and *n* follows a power law:
  (6) $$F\left(n\right)∼n^{\alpha }$$
  where α is the scaling exponent estimated as the slope of the linear regression line fitting the log–log.

The scaling exponent α quantifies the fractal scaling behavior and intrinsic complexity of the signal. For an uncorrelated random signal, α=0.5, while 0.5<α ≤ 1 indicates persistent long-range power–law correlations, and 0<α<0.5 suggests anti-persistent behavior [11].

The DFA method allows for extracting the scaling exponent α as a quantitative nonlinear metric characterizing the inherent complexity and multiscale dynamics of EDA signals across different emotional states based on participant ratings. Additionally, crossover scales marking transitions in scaling behavior can be identified from the log–log plots of $F\left(n\right)$ versus n.

2. Hurst Exponent Estimation:

The Hurst exponent, denoted as H, is a measure of fractal scaling and long-range dependence in a time series. It is related to the scaling exponent α obtained from DFA but provides complementary information about the signal’s fractal properties. The Hurst exponent was estimated using a wavelet-based approach [39].

The method involves the following steps:

The input signal is decomposed into wavelet coefficients using the discrete wavelet transform (DWT). In this implementation, a level 8 wavelet decomposition with ’db5’ (Daubechies 5) wavelet basis was performed.

(7) $$H=\frac{coeffs\left(1\right)}{2}$$

The Hurst exponent ranges from 0 to 1, where *H* = 0.5 indicates an uncorrelated random signal, 0.5 < *H* ≤ 1 suggests persistent long-range correlations (fractal behavior), and 0 < *H* < 0.5 implies anti-persistent behavior [14].

This wavelet-based method for estimating Hurst exponent leverages the multi-resolution analysis capabilities of the wavelet transform and the scaling properties of the detail coefficients. By analyzing the relationship between the variances of the detail coefficients and their corresponding scales, the Hurst exponent can be estimated as a quantitative measure of the signal’s fractal scaling and long-range dependence.

3. Wavelet Entropy:

Wavelet entropy is a measure of signal complexity based on the wavelet transform, which decomposes the signal into different time-frequency components. It quantifies the irregularity and unpredictability of the signal by analyzing the distribution of wavelet coefficients across different scales.

(a) The detrended signal $y_{d}\left[n\right]$ is decomposed using the continuous wavelet transform (CWT):
  (8) $$W_{\psi }\left(a,b\right)=\frac{1}{\sqrt{a}}\int_{-\infty }^{\infty }y_{d}\left(t\right)\psi^{*}\left(\frac{1}{ba}\right)dt$$
  where $\psi \left(t\right)$ is the mother wavelet, a is the scale parameter, and b is the translation parameter.
(b) The wavelet coefficients $W_{\left\{\psi \left(a,b\right)\right\}}$ are normalized to obtain the wavelet energy density:
  (9) $$p_{j}=\frac{1}{N}\sum_{k=1}^{N}{\left|W_{\psi }\left(a_{j},b_{k}\right)\right|}^{2}$$
  where N is the number of translation coefficients at scale $a_{j}$.
(c) The wavelet entropy is then calculated as the Shannon entropy of the wavelet energy density:
  (10) $$E=-\sum_{j=1}^{M}p_{j}log_{2}\left(p_{j}\right)$$
  where M is the number of scales considered.

Higher wavelet entropy values indicate a more complex and irregular signal, while lower values suggest a more regular or predictable signal pattern.

The fractal analysis methods (DFA, Hurst exponent, and wavelet entropy) were applied to the detrended EDA signal segments within each window, providing a comprehensive characterization of the signal’s fractal properties, long-range correlations, and complexity across different time scales. The extracted fractal features were then used as inputs for the emotion classification analysis.

When employing fractal analysis techniques such as DFA, it is crucial to select appropriate parameters to ensure the reliability of the results and their reproducibility by other researchers [27,40]. In the course of our investigation, we discovered that utilizing three-second windows with a 50% overlap proved to be the most effective approach for analyzing the EDA signals. These values were selected following a process of testing different combinations, with the objective of ensuring the capture of rapid changes in emotional responses while maintaining stable measurements. In the case of DFA, particular care was taken to select scaling ranges that avoided both very short time scales (less than 0.5 s), where random noise is stronger, and very long scales (greater than 10 s), where the signal becomes too unpredictable [41]. These parameter choices are consistent with previous studies of physiological signals [42,43,44] and provide a balance between capturing detailed information and obtaining reliable results.

### 2.3. Cross-Correlation Analysis

To investigate the potential relationships and temporal dependencies between the extracted fractal features, specifically the DFA scaling exponents (α) and Hurst exponents (H), and the emotional state labels characterized by arousal and valence scores, cross-correlation analyses were conducted. The cross-correlation functions were computed between the standardized (z-scored) fractal features and the median arousal and valence scores computed for each window. This standardization process ensured that the cross-correlation values were not influenced by the different scales or magnitudes of the time series. The maximum cross-correlation values and their corresponding lags were identified, providing insights into the strength and temporal lag of the correlations between the fractal features and emotional states.

Furthermore, Pearson correlation coefficients were calculated between the fractal features and emotional state labels to quantify the linear associations between these variables, and Granger causality tests were conducted to investigate the presence of potential causal influences between the fractal features and emotional states. These tests aimed to determine whether the past values of one time series could provide statistically significant information for predicting the future values of another time series, beyond the information contained in the past values of the latter.

### 2.4. Statistical Analysis

Prior to the emotion classification analysis, statistical analyses were performed to assess the discriminative power of the extracted fractal features across different emotional states. The normality of the fractal feature distributions (scaling exponents α, Hurst exponents H, wavelet entropy E) for each emotional state was evaluated using the Shapiro–Wilk test [45]. This test helped determine the appropriate statistical methods for subsequent analyses, either parametric or non-parametric, based on the assumption of normality.

Since the fractal features exhibited non-normal distributions, the non-parametric Kruskal–Wallis test was employed to assess if significant differences existed among the emotional states for each fractal feature [46]. The Kruskal–Wallis test is an extension of the Mann–Whitney U test and is suitable for comparing more than two independent groups. A significance threshold of 0.05 was used for this test.

When the Kruskal–Wallis test indicated significant differences among the emotional states for a particular fractal feature, post hoc analyses were conducted to identify the specific pairs of emotional states exhibiting significant differences. The Dunn test with Holm–Bonferroni correction was used for this purpose, as it is a non-parametric multiple comparison procedure that accounts for the familywise error rate (FWER) associated with conducting multiple pairwise comparisons. To control for potential biases arising from the sample size of the dataset, a more stringent significance threshold of 0.005 was adopted for the post hoc Dunn test with Holm–Bonferroni correction [47]. This conservative approach ensured a higher level of confidence in the observed differences and mitigated the risk of false positives.

### 2.5. Machine Learning Analysis

To validate the efficiency of the fractal analysis approach, particularly the fractal features extracted from DFA, in detecting emotional states from EDA signals, we trained and evaluated multiple traditional and state-of-the-art machine learning classifiers for emotional states detection (neutral, amused, bored, relaxed, and scared) using the extracted fractal features as inputs.

#### 2.5.1. Model Selection and Hyperparameter Tuning

A random search method was employed for model selection and hyperparameter tuning [48]. A random search is an efficient technique for exploring the high-dimensional hyperparameter space and identifying the optimal combination of hyperparameters for each machine learning model. This approach was chosen over a grid search due to its computational efficiency and ability to handle complex interactions among hyperparameters.

The following machine learning classifiers were evaluated: Support Vector Machines (SVM), Random Forests (RF), Gaussian Naive Bayes (GNB), Logistic Regression (LR), Gradient Boosting (GBoost), Decision Trees (DT), Extreme Gradient Boosting (XGBoost), K-Nearest Neighbors (KNN). For each classifier, the random search method randomly sampled hyperparameter values from predefined distributions and evaluated the model’s performance using a suitable metric (e.g., balance accuracy, F1-score, recall, precision) on a validation set. This process was repeated for a specified number of iterations, and the hyperparameter combination that yielded the best performance was selected for the final model. For additional validation, we implemented and compared our approach with two state-of-the-art deep learning architectures: a 1D-CNN model with residual connections and a transformer-based model adapted for time-series analysis. We used the same evaluation protocol (LOSO-CV) and preprocessing steps to ensure fair comparison.

#### 2.5.2. Evaluation Procedure

The performance of the trained models was evaluated using a stratified leave-one-subject-out cross-validation (LOSO-CV) scheme. In this approach, the data from one subject was held out as the test set, while the data from the remaining subjects were used for training and validation. This process was repeated until all subjects had been used as the test set once, ensuring a fair and unbiased evaluation of the models’ generalization capabilities. In addition to the classification evaluation metrics, confusion matrices were generated to analyze the patterns of misclassifications among different emotional states.

## 3. Results

**Figure 2:**
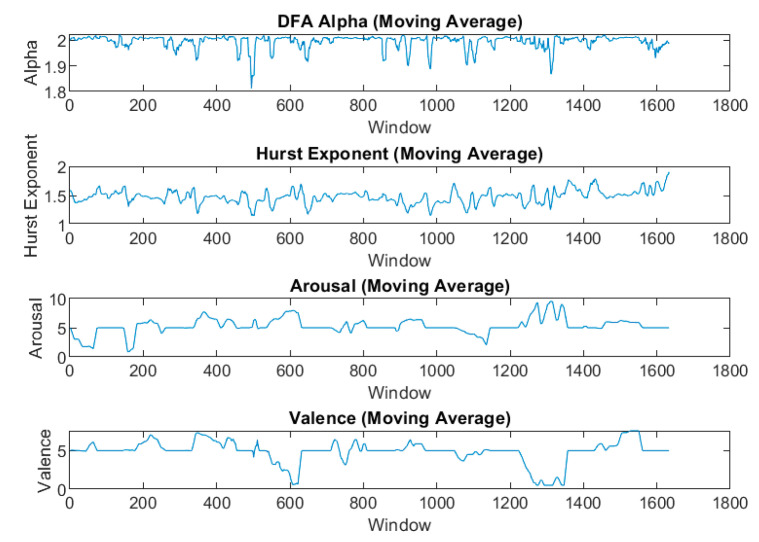
DFA and Hurst exponent moving average following the arousal and valance trends.

Our analysis employed a comprehensive set of fractal and non-linear features extracted from EDA signals. The primary features included the DFA alpha, Hurst exponent, and Box Counting Dimension. We also calculated several wavelet entropy features, specifically the DFA of wavelet entropy, standard deviation of wavelet entropy, minimum and maximum of wavelet entropy, and median of wavelet entropy. To capture more complex dynamics, we extended our feature set to include first-order differences and squared values of all these primary features. This comprehensive approach allowed us to explore both the direct and derived measures of fractal characteristics in the EDA signals. These features were calculated across various window sizes and overlap percentages to identify the most informative configuration for emotion recognition. Figure 2 illustrates how the DFA alpha and Hurst exponent values closely follow the dynamics of both arousal and valence over time for a given subject.

We performed cross-correlation analysis between the fractal features and emotional measures (arousal and valence) across different window sizes and overlap percentages. Table 1 presents the average maximum cross-correlation coefficients for the best-performing configuration.

We identified five key features derived from the wavelet entropy analysis that showed significant differences across the five emotional states: neutral (N), amused (A), bored (B), relaxed (R), and scared (S). These features are the standard deviation of wave entropy (std wave entropy), minimum of wave entropy (min wave entropy), median of wave entropy (median wave entropy), and their squared versions. Table 2 presents these features along with their F-statistic and *p*-values from one-way ANOVA tests.

Post-hoc Tukey HSD tests revealed significant pairwise differences (*p* < 0.005) for most comparisons. Std wave entropy squared and std wave entropy were different for all pairs except B vs. S. Min wave entropy and min wave entropy squared were different between all pairs except B vs. S. Median wave entropy was different between all pairs except A vs. S. Notably, the relaxed (R) state showed highly significant differences (*p* < 2.2 × 10^−16^) from all other emotional states across all features.

**Figure 3:**
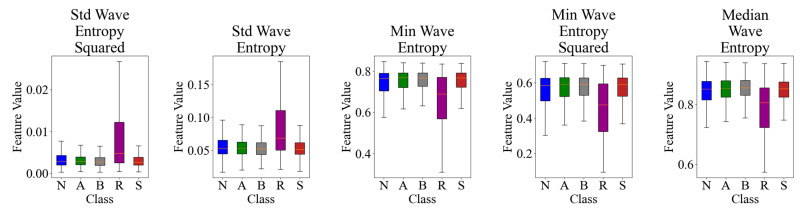
Distribution of top wavelet entropy features across emotional states.

Figure 3 visually represents the distribution of these features across the five emotional states using box plots. The plots illustrate clear differences in feature values between emotional states, particularly for the std wave entropy squared and min wave entropy features. The ‘relaxed’ state often shows a wider range of values, especially in the std wave entropy and its squared version, suggesting higher variability in this emotional state. The median wave entropy appears to differentiate the ‘scared’ state from others, showing generally higher values.

Table 3 presents the performance metrics of the various machine learning algorithms utilized for the classification of emotional states based on the extracted features. The best-performing algorithm was Extreme Gradient Boosting (XGBoost), achieving an F1 score of 0.802 and an accuracy of 84.3%. This illustrates the potential of our fractal analysis features in the context of emotion classification tasks. Random Forests and Gradient Boosting also performed well, suggesting that ensemble methods are particularly effective for this task. Deep learning approaches achieved competitive performance (1D-CNN: 79.8%, transformer: 81.2%)

Our machine learning results demonstrated the usefulness of fractal features for emotion classification. The best-performing algorithm, XGBoost, achieved an accuracy of 84.3% and an F1 score of 0.802. This performance is notably superior to previous methods applied to the CASE dataset, as shown in Table 4.

We performed runtime comparisons between different methods on a high-performance system (Intel Core i9-14900K processor, Intel, Santa Clara, CA, USA, 32 GB RAM). Our method processed one minute of EDA data in 0.18 s, compared to 0.45 s for traditional feature extraction, 1.2 s for graph-based approaches, and 2.3 s for deep learning methods. These results demonstrate that our fractal analysis approach not only achieves superior classification performance but also maintains computational efficiency, making it particularly suitable for real-time applications and resource-constrained devices.

## 4. Discussion

Our study employed a comprehensive set of fractal analysis features to analyze EDA signals in relation to emotional states. Our analysis revealed that a window size of 3 s with 50% overlap provided the best results in terms of correlation between fractal features and emotional measures. This configuration likely represents an optimal balance between capturing short-term fluctuations and maintaining sufficient data for robust fractal analysis. The 3-second window aligns with previous research suggesting that emotional responses in EDA occur on a timescale of 1–4 s [6,38], while the 50% overlap allows for smooth tracking of temporal changes.

The strong correlations observed between our fractal features and both arousal and valence (e.g., wavelet entropy showing correlations of 0.76 and 0.71 with arousal and valence, respectively) are particularly noteworthy. These correlations suggest that fractal features capture meaningful information about both dimensions of emotion, challenging the traditional view that EDA primarily reflects arousal [49,50,51]. This has significant implications for affective computing and emotion-aware systems [8]. The ability to infer both arousal and valence from a single physiological signal (e.g., EDA) using fractal analysis could greatly simplify and improve emotion recognition systems. It suggests the possibility of developing real-time emotion labeling methods that do not require subjective input from the participant, potentially opening new applications in fields such as human–computer interaction, mental health monitoring, and personalized user experiences.

These strong correlations between fractal features and both arousal (r = 0.76) and valence (r = 0.71) suggest that EDA contains more emotional information than previously thought. This challenges the traditional view of EDA as primarily an arousal indicator. This relationship may be explained by the complex interaction between sympathetic nervous system activation patterns and emotional states, where different emotional contexts create distinct fractal signatures in the EDA signal. This finding aligns with recent research on the multidimensional nature of autonomic responses to emotions [13,49].

The high discriminative power of our features for the relaxed state is particularly noteworthy. This suggests that relaxation, often considered a baseline or neutral state in many studies, may have a distinct physiological signature. This finding has implications for how we conceptualize and measure baseline states in emotion research [52]. The substantial improvement over previous methods (an 8.8 percentage point increase in accuracy and a 0.117 increase in F1 score compared to EDA-Graph features) underscores the power of fractal analysis in capturing emotion-relevant information from EDA signals (Table 4). This improvement is particularly significant given the challenging nature of emotion recognition from physiological signals and the relatively subtle differences between some emotional states.

The superior performance of ensemble methods (XGBoost, Random Forests, Gradient Boosting) in our analysis suggests that the relationships between fractal features and emotional states are complex and non-linear. These sophisticated algorithms can capture intricate patterns and interactions between features that simpler models may miss. This aligns with the inherent complexity of emotion-related physiological processes, which involve interactions between multiple physiological systems and can vary considerably between individuals [23,37,38,53].

The use of fractal analysis in this context is particularly powerful as it captures the complex, multi-scale dynamics of physiological signals that may not be apparent in traditional time-domain or frequency-domain analyses [43,54]. This approach aligns with the growing recognition that physiological systems exhibit complex, non-linear behaviors that require sophisticated analytical tools to fully understand [55]. The DFA, Hurst exponent, and wavelet entropy emerged as particularly informative features. DFA, which quantifies long-range correlations in non-stationary time series, has been shown to be sensitive to changes in autonomic nervous system activity associated with different emotional states [56]. In our study, the DFA alpha values showed significant differences across emotional states, suggesting that the temporal structure of EDA fluctuations carries important information about emotional processes.

The Hurst exponent, indicating the degree of self-similarity in the signal, provides insight into the persistence or anti-persistence of EDA fluctuations. Our findings showed that the squared Hurst exponent was even more discriminative than the raw Hurst exponent, highlighting the non-linear nature of emotion-related EDA dynamics. This aligns with previous research suggesting that emotional processes can induce changes in the fractal scaling of physiological signals [7]. Wavelet entropy, which quantifies the degree of disorder in the time-frequency domain, emerged as a particularly powerful feature in our analysis. The strong performance of wavelet entropy-derived features (e.g., DFA of wavelet entropy, max of wavelet entropy) suggests that emotions modulate the complexity of EDA signals across multiple time scales. This multi-scale approach to signal complexity has been shown to be sensitive to subtle changes in physiological state that may not be captured by single-scale measures [57].

The effectiveness of squared versions of our features in differentiating emotional states underscores the importance of non-linear transformations in emotion recognition. This finding supports recent work suggesting that non-linear transformations of physiological measures can enhance their sensitivity to subtle emotional changes [58]. It highlights the complex, non-linear nature of emotion-related physiological processes and suggests that future work in affective computing should consider such non-linear feature transformations.

Furthermore, our results challenge the traditional view of EDA as primarily an indicator of arousal. The ability of wavelet entropy features to differentiate between states that are similar in arousal (e.g., neutral vs. bored) or valence (e.g., amused vs. scared) suggests that these features are capturing more nuanced aspects of emotional physiology. This aligns with contemporary emotion theories that view emotions as complex, multi-component phenomena rather than simple points in a two-dimensional arousal–valence space [22,23,37].

A detailed analysis of our classification results reveals important patterns in emotion recognition performance. The model showed the highest precision for high-arousal states like ’scared’ (0.89) and distinct valence states like ’relaxed’ (0.86), suggesting that these emotional states produce more distinctive fractal signatures in the EDA signal. Neutral states showed relatively lower precision (0.78), which aligns with previous findings about the challenges of detecting baseline emotional states. The confusion matrix reveals minimal misclassification between opposite emotional states (e.g., relaxed vs. scared), while showing some expected confusion between adjacent emotions in the circumplex model. This pattern supports the physiological validity of our approach, as it reflects the known continuous nature of emotional states. These results demonstrate that fractal analysis can effectively capture the nuanced differences between emotional states while maintaining robustness to individual variations in EDA responses.

The superior performance of our fractal analysis approach becomes particularly evident when compared to previous methods applied to the CASE dataset (Table 4). Our approach achieved an accuracy of 84.3% and an F1 score of 0.802, representing a substantial improvement over existing methods. In our recent study using EDA-Graph, which utilized graph signal processing, we achieved 75.5% accuracy and an F1 score of 0.68. The fractal analysis approach achieved a significantly higher performance with 84.3% accuracy and an F1 score of 0.802. This represents an improvement of 8.8 percentage points in accuracy and 0.122 in F1 score using fractal analysis.

EDA-Graph already significantly outperformed previous approaches like isaxEDA (69.0% accuracy, 0.65 F1), netEDA (70.0% accuracy, 0.64 F1), and topEDA (69.0% accuracy, 0.63 F1) [22]. This suggests that fractal analyses may be capturing fundamental aspects of emotional physiology that even sophisticated graph-based approaches miss. It is worth noting that both approaches surpass traditional EDA feature analysis (68.4% accuracy, 0.56 F1) by a considerable margin, confirming the value of advanced non-linear analysis methods in emotion recognition. Our method’s superior performance (84.3% accuracy vs. 75.5% for EDA-Graph) can be attributed to several factors: (1) the ability of fractal features to capture multi-scale dynamics missed by traditional approaches; (2) the complementary nature of our feature set combining DFA, Hurst exponent, and wavelet entropy; and (3) the robustness of fractal analyses to individual variations in EDA responses. The improvement over deep learning approaches (58.7% for few-shot learning) suggests that our engineered features better capture the underlying physiological patterns of emotional states.

The results show that while these deep learning approaches achieved competitive performance (1D-CNN: 79.8%, transformer: 81.2%), our fractal analysis method (84.3%) maintained superior accuracy while requiring significantly less computational resources and training data. While our method outperformed existing approaches, including a deep learning model [37], we acknowledge the need for more extensive comparisons with recent deep learning architectures. However, it is worth noting that our fractal analysis approach offers several advantages over deep learning methods: (1) it requires significantly less training data, (2) it provides interpretable features that directly relate to physiological processes, and (3) it is computationally more efficient. Future work will include comprehensive comparisons with transformer-based architectures and other recent deep learning approaches while considering these trade-offs in practical applications.

We believe that the significant performance improvement is due to several factors. First, the multiscale nature of our fractal analysis approach allows it to capture both fine-grained and broader patterns in EDA signals. Second, the combination of DFA with wavelet entropy analysis provides complementary information about signal complexity and temporal structure. Also, the ability of fractal features to capture nonlinear dynamics may be missed by traditional analysis methods or even more recent approaches such as graph signal processing. Finally, compared to deep learning approaches, fractal analysis captures fundamental patterns in EDA signals that remain relatively consistent across individuals, unlike deep learning methods that may require much more data to find these patterns [59].

While our results show significant improvements over previous methods, we should note some important limitations. Our study used data from 30 participants in the CASE dataset, which might not represent all the ways different people show emotions through their EDA signals [60]. This becomes particularly important when thinking about real-world applications, where factors like age, culture, and personal differences might affect how people’s bodies respond to emotions [61]. Studies with larger and more diverse groups of participants, especially in natural settings rather than laboratories, would help confirm how well our method works across different populations and situations [1].

Furthermore, while we have shown strong correlations and classification performance, the causal mechanisms linking fractal properties of EDA to emotional states remain to be fully elucidated. Emotional states are complex, and there is potential for overlapping in the physiological responses between emotional states. There is a possibility that confounding variables not accounted for in the analysis may have influenced the results. For instance, the observation that bored and fearful states did not differ significantly in some characteristics suggests the need for further investigation of how electrodermal activity (EDA) dynamics might reflect more nuanced emotional dimensions.

Future studies should explore the potential for real-time emotion recognition using sliding window fractal analysis of EDA and investigate how fractal features of EDA interact with other physiological signals (e.g., heart rate variability, respiratory patterns) in emotion recognition tasks. The proposed system has several potential real-world applications. The EDA data collection could be implemented using a wearable device that continuously runs the emotion detection model, enabling mental health monitoring, allowing for early detection of emotional distress patterns; human–computer interaction, allowing devices to adapt to users’ emotional states in real time; educational technology, helping to identify student engagement and stress levels; and clinical assessment, providing objective measures of emotional regulation in therapeutic settings. The computational efficiency of our approach makes it particularly suitable for implementation in wearable devices with limited processing power.

Future work will focus on validating our method across different EDA datasets, particularly those with diverse demographic representations and varying experimental conditions. Some candidate datasets for this extension include DEAP [62], AMIGOS [63], WESAD [64], and the Mixed Emotion Dataset [65], which offer different experimental paradigms and population characteristics. This multi-dataset validation will help establish the robustness and generalizability of our fractal analysis approach across different contexts and populations.

## 5. Conclusions

The present study demonstrates the potential of fractal analysis in the field of emotion recognition, specifically in the context of EDA signals. The capture of complex, multi-scale dynamics through fractal features provides a rich source of information that significantly outperforms traditional approaches. The potential to infer both arousal and valence from EDA using these methods offers new avenues for the development of emotion-aware systems and affective computing applications. As we continue to elucidate the intricate relationships between physiological dynamics and emotional experiences, fractal analysis emerges as a promising tool in our methodological arsenal.

## Figures and Tables

**Table 1:** Average maximum cross-correlation coefficients (window size: 3 s, overlap: 50%).

Feature	Arousal	Valence
DFA Alpha	0.72 (*p* < 0.001)	0.68 (*p* < 0.001)
Hurst Exponent	0.69 (*p* < 0.001)	0.65 (*p* < 0.001)
Wavelet Entropy	0.76 (*p* < 0.001)	0.71 (*p* < 0.001)

**Table 2:** Top five features with significant differences between emotional states.

Feature	F-Statistic	*p*-Value
Std Wave Entropy Squared	237.45	*p* < 0.001
Std Wave Entropy	225.31	*p* < 0.001
Min Wave Entropy	218.76	*p* < 0.001
Min Wave Entropy Squared	210.89	*p* < 0.001
Median Wave Entropy	205.63	*p* < 0.001

**Table 3:** Machine learning classification results.

Algorithm	F1 Score	Accuracy	Precision	Recall
SVM	0.789	82.1%	0.795	0.783
RF	0.798	83.5%	0.810	0.786
GNB	0.756	79.2%	0.762	0.750
LR	0.775	81.0%	0.780	0.770
GB	0.795	83.1%	0.803	0.787
DT	0.768	80.5%	0.775	0.761
XGBoost	**0.802**	**84.3%**	**0.815**	**0.789**
KNN	0.771	80.8%	0.778	0.764

**Table 4:** Comparative analysis of previous methods and current approach.

Method	Accuracy	F1 Score	Description
Few-Shot Learning [37]	58.7%	0.48	Deep Siamese network approach for emotion classification
IsaxEDA [22]	69.0%	0.65	Symbolic approximation-based method
comEDA [22]	67.0%	0.55	Complexity-based analysis approach
topEDA [22]	69.0%	0.63	Topological analysis method
netEDA [22]	70.0%	0.64	Network theory-inspired approach
EDA-Graph [23]	75.5%	0.68	Graph signal processing approach
1D-RCNN	79.8%	0.762	1D-CNN model with residual connections
Transformer Based	81.2%	0.792	Transformer-based model adapted for time-series analysis
Traditional EDA Features (BEC)	68.4%	0.56	Classic feature extraction with bagging classifier
Fractal Analysis (Current Study)	80.8%	0.771	DFA and wavelet entropy-based approach

## Data Availability

The data presented in this study are openly available in GitLab at https://gitlab.com/karan-shr/case_dataset, access date 5 August 2024.
